# Dissecting the genetic architecture of root-related traits in a grafted wild *Vitis berlandieri* population for grapevine rootstock breeding

**DOI:** 10.1007/s00122-023-04472-1

**Published:** 2023-10-14

**Authors:** Louis Blois, Marina de Miguel, Pierre-François Bert, Nathalie Ollat, Bernadette Rubio, Kai P. Voss-Fels, Joachim Schmid, Elisa Marguerit

**Affiliations:** 1 EGFV, Bordeaux Sciences Agro, INRAE, ISVV, Univ. Bordeaux, 33882 Villenave d’Ornon, France; 2 Department of Grapevine Breeding, Geisenheim University, Von Lade Str. 1, 65366 Geisenheim, Germany

## Abstract

In woody perennial plants, quantitative genetics and association studies remain scarce for root-related traits, due to the time required to obtain mature plants and the complexity of phenotyping. In grapevine, a grafted cultivated plant, most of the rootstocks used are hybrids between American *Vitis* species (*V. rupestris*, *V. riparia*, and *V. berlandieri*). In this study, we used a wild population of an American *Vitis* species (*V. berlandieri*) to analyze the genetic architecture of the root-related traits of rootstocks in a grafted context. We studied a population consisting of 211 genotypes, with one to five replicates each (*n* = 846 individuals), plus four commercial rootstocks as control genotypes (110R, 5BB, Börner, and SO4). After two independent years of experimentation, the best linear unbiased estimates method revealed root-related traits with a moderate-to-high heritability (0.36–0.82) and coefficient of genetic variation (0.15–0.45). A genome-wide association study was performed with the BLINK model, leading to the detection of 11 QTL associated with four root-related traits (one QTL was associated with the total number of roots, four were associated with the number of small roots (< 1 mm in diameter), two were associated with the number of medium-sized roots (1 mm < diameter < 2 mm), and four were associated with mean diameter) accounting for up to 25.1% of the variance. Three genotypes were found to have better root-related trait performances than the commercial rootstocks and therefore constitute possible new candidates for use in grapevine rootstock breeding programs.

**Supplementary Information:**

The online version contains supplementary material available at 10.1007/s00122-023-04472-1.

## Introduction

Climate change is driving a need to adapt to new environmental conditions through many approaches, including the modification of plant material. The roots of the plant manage its nutrient and water absorption. This organ therefore plays a major role in plant physiology and productivity and constitutes a very good target in breeding for plant adaptation (Voss-Fels et al. 2018). The root system has been identified as a target for breeding for abiotic stress tolerance and yield in cereals (Meister et al. 2014; Maqbool et al. 2022). However, the growing conditions in annual crops must also be taken into account when breeding root traits, to optimize root architecture and root system carbon allocation (Lynch 2018).

High levels of phenotypic variability have been observed for root-related traits in *Arabidopsis* (Pacheco-Villalobos and Hardtke 2012) and in annual crops, but few field trials have been performed due to the complexity of root phenotyping in field conditions (Deja-Muylle et al. 2020; Maqbool et al. 2022). There have also been few studies in perennial plants with potentially high levels of root phenotypic variability, such as *Picea* species (Nielsen 1992), poplar (Wullschleger et al. 2005; Krabel et al. 2015; Sun et al. 2019), and other angiosperms (Seago and Fernando 2013). Accordingly, the genetic basis of root-related traits has been explored in several annual species, such as maize (Hochholdinger and Tuberosa 2009; Pace et al. 2015; Zaidi et al. 2016; Bray and Topp 2018; Sanchez et al. 2018) and rice (Courtois et al. 2009; Mai et al. 2014; Biscarini et al. 2016; Phung et al. 2016; Zhao et al. 2018), but much less is known about perennial species, such as ryegrass (Sun et al. 2019) and woody species (Nielsen 1992; Wullschleger et al. 2005; Krabel et al. 2015; Sun et al. 2019).

Grapevine is a major horticultural crop around the world. Since the decimation of grapevine crops due to the phylloxera crisis in Europe, grapevine has been cultivated as a grafted crop. Most of the rootstocks used are hybrids between the American *Vitis* species *V. rupestris*, *V. berlandieri*, and *V. riparia* (Galet 1988). The grafted nature of modern grapevine crops renders the rootstock a major target for root system breeding. Thanks to the interactions that occur between the scion and the rootstock in grapevine, the rootstock is a precious tool for grapevine adaptation (Ollat et al. 2016). The use of *Vitis berlandieri* as a rootstock has been shown to provide a high tolerance to limestone soils and drought on the scion as well as phylloxera protection (Boubals 1966; Galet 1988). However, the rooting and grafting performances of *V. berlandieri* are generally limiting for its direct use as a rootstock, and it is usually crossed with other American species to obtain hybrids, which are widely used in vineyards: 1103 Paulsen (1103P), 110 Richter (110R), Fercal, Selection Oppenheim 4 (SO4), and Gravesac (FranceAgrimer, 2018).

Most studies aiming to decipher the genetic architecture of relevant traits for rootstocks have been based on controlled crosses and QTL analysis (Xu et al. 2008; Zhang et al. 2009; Clark et al. 2018; Henderson et al. 2018; Smith et al. 2018a, 2018b). Moreover, only a few of these studies used grafted grapevines (Marguerit et al. 2012; Bert et al. 2013; Tandonnet et al. 2018). Most genome-wide association studies (GWAS) in grapevine have been restricted to *Vitis vinifera* (Fournier-Level et al. 2009; Emanuelli et al. 2010; Myles et al. 2011; Migicovsky et al. 2017; Marrano et al. 2018; Flutre et al. 2019, 2020) or *Vitis spp.* (Yang et al. 2017; Zhang et al. 2017; Laucou et al. 2018; Guo et al. 2019; Liang et al. 2019; LaPlante et al. 2021; Trenti et al. 2021; Wang et al. 2021) germplasm collections. GWAS have never been performed for wild *Vitis* populations or grafted grapevines. Moreover, previous studies have targeted berry traits, water deficit tolerance and cold tolerance, but not the root system.

Broad genetic diversity has been observed in American *Vitis* species (Péros et al. 2021), but the various genetic backgrounds have yet to be explored. In association studies, the high level of genetic diversity in wild populations and the large numbers of recombination events occurring over many generations can be used to narrow down the positions of loci tightly linked to the trait of interest. The use of such studies to investigate root-related traits can, therefore, improve our understanding of the genetic basis of these traits in a perennial species and identify new beneficial alleles for improving grapevine rootstock breeding programs. However, the large number of individuals required to detect significant associations constitutes a real challenge for root-related traits in grapevine, due to the difficulty phenotyping this underground plant organ in a perennial species.

The aims of this study were: (i) to characterize root-related traits in a wild American *Vitis* species used for grafting, (ii) to perform GWAS for root-related traits in a wild grapevine genetic background (*V. berlandieri*) and (iii) to compare the root-related trait performances of wild genotypes with those of commercial rootstocks. Our results reveal the diversity for root-related traits present in the wild *V. berlandieri* population. In addition, GWAS highlighted promising markers associated with root-related traits in a wild population.

## Materials and method

### Plant material

The plant material used in this study consisted of 286 genotypes originating from 78 mother plants of wild *V. berlandieri* collected from the Edwards Plateau in Texas, USA (see Blois et al. 2023 for further details). This population is considered as pure *V. berla*ndieri due to the classical ampelographic detection and a molecular one. Moreover, the extreme environmental conditions of the region limit any development of other species. All the plants were used as rootstocks, onto which we grafted *Vitis vinifera* Riesling (clone 24–209 for two consecutive years, 2019 and 2020). Not all of the genotypes were represented every year in the final population, the genotypes present in a given year depending on the success of grafting (Table S1). The commercial rootstocks 110R (*V. berlandieri* cv. Boutin B × *V. rupestris* cv. Martin), SO4 (*V. berlandieri* Rességuier 2 ×* V. riparia* Gloire de Montpellier), Börner (*V. riparia* 183 G × *V. cinerea* Arnold) and 5BB (*V. berlandieri* Rességuier 2 ×* V. riparia* Gloire de Montpellier) (2020 only) were added to the population as control genotypes. The aim was for each genotype to be represented in at least five replicates, where possible. We obtained 181 genotypes (510 individuals) in 2019, and 144 genotypes (336 individuals) in 2020. In total, 211 genotypes were represented, as 846 individuals and 4 commercial rootstocks (35 individuals). Phenotyping was performed the year after grafting. The 2020 plant pool therefore resulted from the grafting performed in 2019 and the 2021 plant pool corresponding to the grafting performed in 2020. Grafting was performed at the Institute of Grapevine Breeding in Geisenheim, Germany. Cuttings were collected in the field and cut into 20 cm-long pieces in February. All cuttings were stored in wet bags in a cold room (3 °C) after Beltanol treatment to prevent fungal contamination. Grafting was performed by a classical mechanical procedure, with an omega graft, in March 2019 and 2020. During grafting, a special attention has been paid to match the diameters of the two partners. The grafted material was placed in a warm room for one month, after which callus quality was evaluated for removal of plants with a low graft quality. The plants were then grown in gray rectangular plastic containers for one month in a mixture of potting soil (2/3, from Einheitserde “classic”) and peat (1/3). Then, the plants with good root and vegetative shoot growth were individually potted (black plastic pots of 2L) in a potting soil medium and grown in the absence of limiting conditions (no symptom of water deficit or nutrient deficiency) until November in Geisenheim, Germany. The plants were grown in a greenhouse and pots were placed on a table. The vegetative part was trained as a staked single shoot. Plant protection products were applied every three weeks from May to September against Peronospora and Oidium.

### Root phenotyping

Plants were pruned after 2 nodes, potted out, washed with pressurized water and stored in a cold room (3 °C) at the end of November. The following year (May 2020 and January 2021 for plants grafted in 2019 and 2020, respectively), roots were cut 4 cm below the collar. Scions and rootstocks were measured with a semi-automatic caliper to determine their diameter and weighed. Primary roots were counted and all diameters were measured with the semi-automatic caliper. Roots were sorted according to their diameter. Those with a diameter of less than 1 mm were considered to be *small* roots, those with a diameter of 1–2 mm were considered to be *medium-sized* and those with a diameter of more than 2 mm were considered to be *large* roots. The entire root system was dried in a drying oven at 80 °C for three days and weighed. The traits measured were root dry weight (RDW), the total number of roots (Tot_Root_NB), total root diameter (Tot_diam), calculated as the sum of all primary root diameters for a single plant, average diameter (Av_Diam), calculated as the mean diameter of all primary roots from the same plant, the number of small roots (NB_Small, diameter < 1 mm), the number of medium-sized roots (NB_Medium, 1 mm >  diameter < 2 mm), the number of large roots (NB_Large, diameter > 2 mm), the proportion of small roots (Prop_Small), the proportion of medium-sized roots (Prop_Medium, I), the proportion of large roots (Prop_Large), scion diameter (SD), rootstock diameter on the thinner and wider sides (RSD_1 and RSD_2, respectively) and the weight of the woody part (PW).

### Genotyping-by-sequencing (GBS) data and SNP selection

The GBS data were obtained by sequencing accession PRJNA886619 (Blois et al. 2023). Only genotypes for which phenotypic data were available were used for SNP filtering (*n* = 211). We used the protocol described by Blois et al. (2023) for SNP calling. VCFtools (Danecek et al., 2011) was used for filtering on minimum depth of 3, maximum missing data of 0.9, minor allele frequency of 0.05 and a minimum mean depth of 20. In total, 102,296 SNPs were retained and 206 genotypes with less than 70% missing data were analyzed.

### Statistical analysis

Statistical analysis was performed without the commercial rootstocks. The density curve was calculated with the stat_lab function of the ggdist package of R with an adjustment of 0.01. The correlations between traits were explored with Pearson’s correlation test. Principal component analysis (PCA) was performed with RDW, Tot_Diam, AV_Diam, Tot_Root_NB, NB_Small, NB_Medium, and NB_Large. The scion and rootstock diameters and plant weight were not considered in this analysis because these traits are not root-related. The proportion of roots in each size class was correlated with the number of roots in each size class. These traits were therefore excluded from the PCA to ensure that the results obtained were not unbalanced. The control commercial rootstocks were considered as additional individuals but were not included in the calculation of coordinates. Missing data were imputed as the mean value for the trait. Only the 30 genotypes with the highest coordinate on each axis were labeled.

Best linear unbiased predictors (BLUPs) were estimated for each trait, to obtain phenotypic values corrected for environmental variability from the genotype replicates and both years of the experiment. The model used was selected according to BIC (best indicator criterion) information

1 $$P_{ghi} = \mu + G_{{\text{g}}} + Y_{h} + W_{i} + \varepsilon_{ghi}$$

where *P*_*ghi*_ is the phenotypic value for genotype (*G*_g_), year of measurement (*Y*_*h*_) and the weight of the plant (*W*_*i*_) after shoot and root pruning. *ε*_*ghi*_ is the residual variance. Plant weight was used to correct phenotypic data as it affects plant carbon reserves and thus root growth. Genotype was considered as a random effect in the model, to obtain a variance–covariance matrix for the calculation of broad-sense heritability (*H*^2^). All the others factors were considered as fixed effects in the model.

The broad-sense heritability of traits was calculated according to Eq. (2),

2 $$H^{2} = \frac{{\sigma_{{\text{g}}}^{2} }}{{\sigma_{{\text{g}}}^{2} + (\sigma_{e}^{2} /{\text{nrep}})}}$$

where *H*^2^ is the broad-sense heritability of the trait, $\sigma_{g}^{2}$ is the variance explained by the genotype effect and $\sigma_{e}^{2} /{\text{nrep}}$ is the residual variance extracted from the model divided by the mean number of replicates per genotype in the population.

These models were calculated in R (R Core Team 2023), with the lme4 package (Bates et al. 2015).

### GWAS

We assessed the estimated genetic value of genotypes for each trait while avoiding the variance shrinkage associated with predictive models, by calculating the best linear unbiased estimate (BLUE) with a model similar to Eq. 1 but with all the factors treated as fixed effects in a generalized linear model. The intercept of each genotype was then used as a new phenotypic value in the GWAS. With this procedure, we used only one phenotypic value per genotype for the two years of experiment.

For GWAS, the BLINK model was used in GAPIT3 (Wang and Zhang 2021) with default settings, implementation by major allele and MAF > 0.05 filtration. We retained 87,589 SNPs for further analysis. Population structure was considered as a covariate with *K* = 2 (Blois et al. 2023). Kinship was derived from pseudo-QTN information, directly from BLINK. Bonferroni correction was applied to the significance thresholds, which were set at 0.05/*n* and 0.01/*n*, where “*n*” is the number of markers used. The variance explained by significant SNPs were estimated from BLINK results in GAPIT with a mixed linear model.

The genes linked to significant markers were obtained by comparison with the annotated *V. berlandieri* genome, with a window corresponding to the extent of linkage disequilibrium (LD) (physical distance reached for *r*^2^ = 0.2 according to Hill and Weir, (1988)) on the corresponding chromosome (mean linkage disequilibrium decay of 2.2 kb, as described in Blois et al. 2023). This procedure made it possible to obtain genes linked to all significant markers except chr5_19758975. In this case, the two flanking genes of the marker region were considered. Gene functions were defined according to information available from UniProt (The UniProt Consortium 2021).

## Results

### Genetic variability of root-related traits

**Fig. 1:**
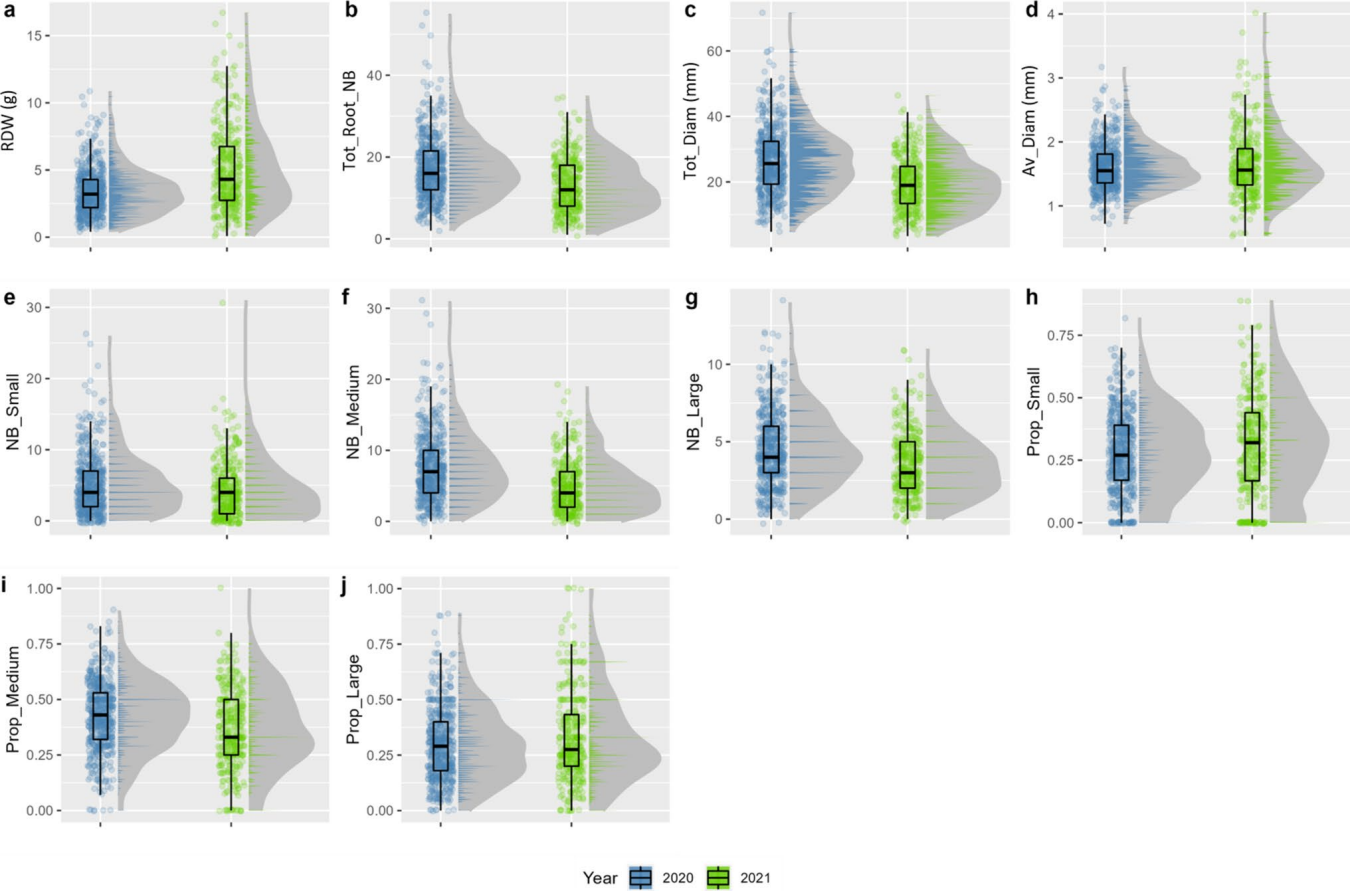
Boxplots and distributions of root-related traits measured in 2020 (blue) and 2021 (green). For each trait, a boxplot is shown on the left and a bar plot distribution on the right, with a density curve indicated in gray. The density curve was calculated with the stat_lab function of the ggdist package in R with an adjustment of 0.01. The traits shown are root dry weight (RDW, **A**), total root number (Tot_Root_NB, **B**), total diameter (Tot_diam, **C**), mean diameter (Av_Diam, **D**), the number of small roots (NB_Small, diameter < 1 mm, **E**), the number of medium roots (NB_Medium, 1 mm <  diameter < 2 mm, **F**), the number of large roots (NB_Large, diameter > 2 mm, **G**), the proportion of small roots (Prop_Small, **H**), the proportion of medium roots (Prop_Medium, **I**), and the proportion of large roots (Prop_Large, **J**) (color figure online)

Phenotypic variability was observed for root traits in the *V. berlandieri* population in 2020 and 2021 (Fig. 1). RDW was higher in 2021 than in 2020; Tot_Root_NB, Tot_Diam, and the number roots in each size class were lower in 2021 than in 2020 (Table S2). RDW ranged from 0.4 to 10.9 g in 2020 and from 0.1 to 16.7 g in 2021, with a higher mean in 2021 than in 2020 (35% lower in 2020) (Table S2). PW, SD and RSD were very similar over the two years of experiment, with a mean PW of 27.6 g and 27.0 g, a mean SD of 4.9 mm and a mean RSD_1/RSD_2 of 7.2/8.4 mm and 7.3/8.3 mm in 2020 and 2021 respectively. Tot_Root_NB in the *V. berlandieri* population was 32% higher in 2020 (mean = 17.2) than in 2021 (mean = 13.0). Mean Tot_Diam was higher in 2020 (26.4 mm) than in 2021 (19.6 mm), but mean Av_Diam was similar in 2021 (1.7 mm) and 2020 (1.6 mm). The numbers of roots in each size class were greater in 2020 than in 2021 with mean values of 5.1, 7.6, and 4.5 for the number of small, medium-sized and large roots, respectively, in 2020 and 4.4, 5.0, and 3.6 for the numbers of small, medium-sized and large roots, respectively, in 2021. The additional roots observed in 2020 were evenly distributed between the three diameter-based classes (*small*, *medium-sized* and *large*), with the same proportion for each class of roots in the 2 years of measurement (0.3, 0.4 and 0.3 for small, medium-sized and large roots, respectively).

The genetic coefficient of variation (CVg) was high, except for traits not related to genetic performance (SD and RSD). Excluding SD and RSD, CVg ranged from 0.15 for the proportion of *medium-sized* roots to 0.45 for the number of *medium-sized* roots (Table 1). For all traits, heritability was moderate to high, ranging from 0.36 for the proportion of *medium-sized* roots to 0.82 for the number of roots.

**Table 1:** Summary of root traits in 2020 and 2021 (*H*^2^ is the broad-sense heritability of traits for the 2 years of the experiment calculated from genetic models, CVg is the coefficient of variation based on BLUP values from the same model)

	*H*^2^	CVg	Min.	Max.	Mean	SD
RDW (g)	0.71	0.34	0.1	16.7	4.0	2.5
SD (mm)	0.45	0.06	2.3	7.3	4.7	0.7
RSD_1 (mm)	0.53	0.04	5.0	11.2	7.3	0.9
RSD_2 (mm)	0.62	0.04	5.9	11.8	8.3	1.0
Tot_Root_NB	0.82	0.32	1.0	55.0	15.6	7.6
Tot_Diam (mm)	0.73	0.22	1.4	71.7	23.3	9.5
Av_Diam (mm)	0.47	0.21	0.3	10.0	1.7	0.9
NB_Small	0.61	0.44	0	31.0	4.9	4.1
NB_Medium	0.79	0.45	0	31.0	6.6	4.5
NB_Large	0.56	0.25	0	14.0	4.2	2.3
Prop_Small	0.48	0.27	0	0.9	0.3	0.2
Prop_Medium	0.36	0.15	0	1.0	0.4	0.2
Pop_Large	0.64	0.32	0	1.0	0.3	0.2

**Fig. 2:**
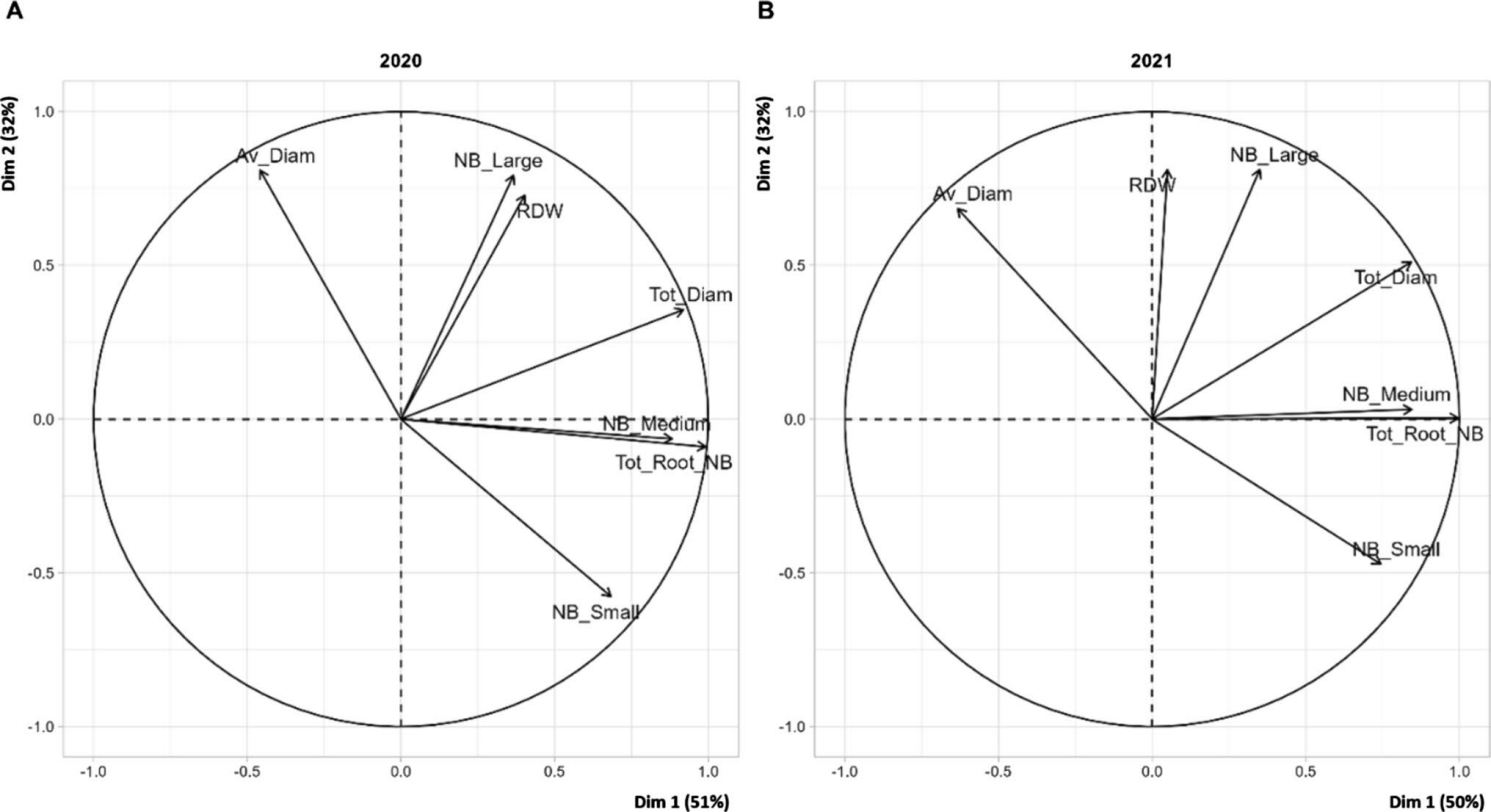
Graph of variables obtained from the PCA analyses in 2020 (**A**) and 2021 (**B**). Commercial rootstock performances were not considered for the calculation of coordinates

**Fig. 3:**
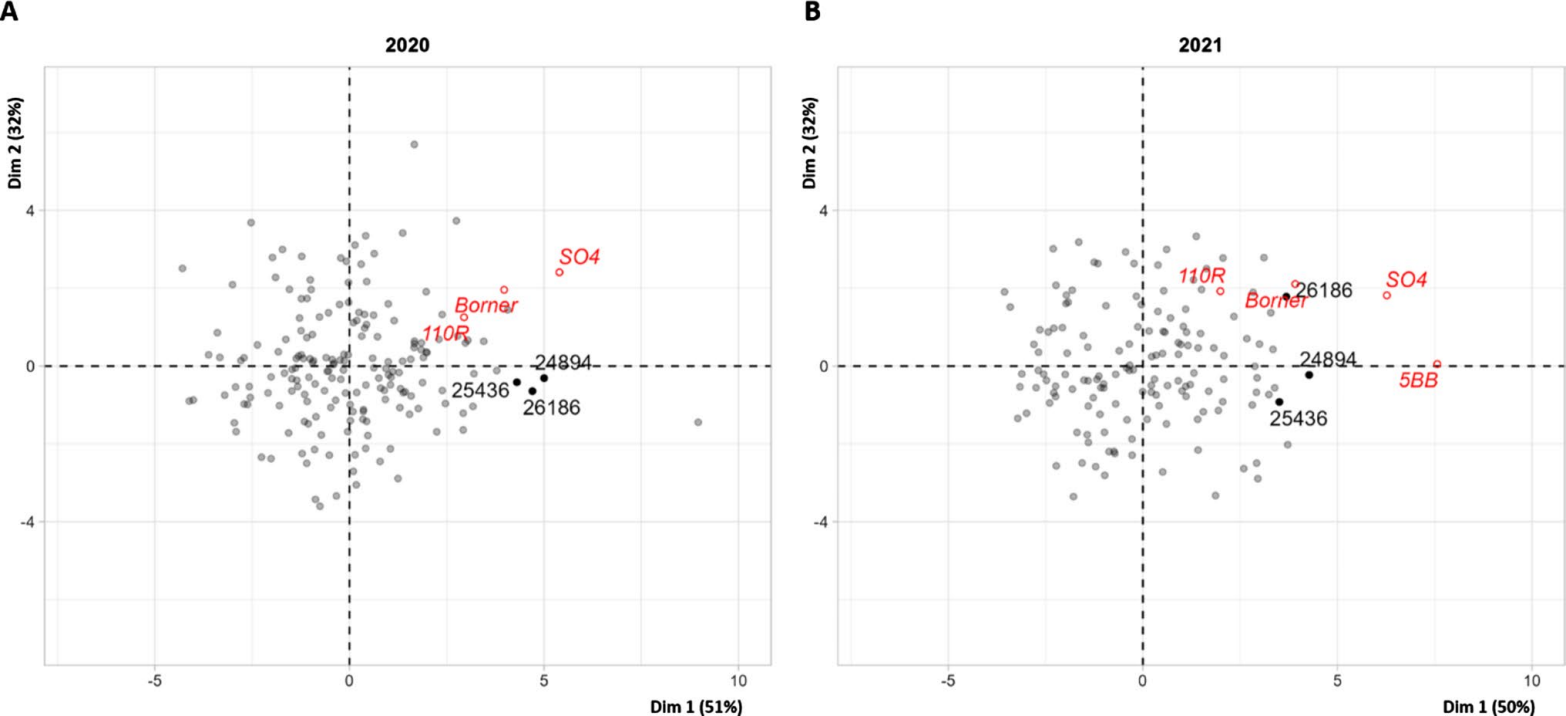
Graph of individuals obtained from the PCA analyses in 2020 (**A**) and 2021 (**B**). Commercial rootstocks (110R, Börner, 5BB and SO4) are indicated in red as additional individuals. Numbers 24894, 25,436 and 26,186 indicate individuals with extreme performances for root-related traits similar to those of commercial rootstocks over the two years of the experiment

The variables were organized similarly in 2020 and 2021 and the first two principal components explained 80% of the variability of traits (Fig. 2). RDW and the number of large roots were correlated, as were RN and the number of medium-sized roots. The panel of genotypes studied was not identical for the two years. It was therefore very difficult to compare individual coordinates. However, a small number of genotypes with more extreme coordinates, close to those of commercial rootstocks, are labeled on Fig. 3A, B. The same three genotypes (26,186, 25,436, 24,894) were labeled in both years and had similar coordinates in both years.

**Fig. 4:**
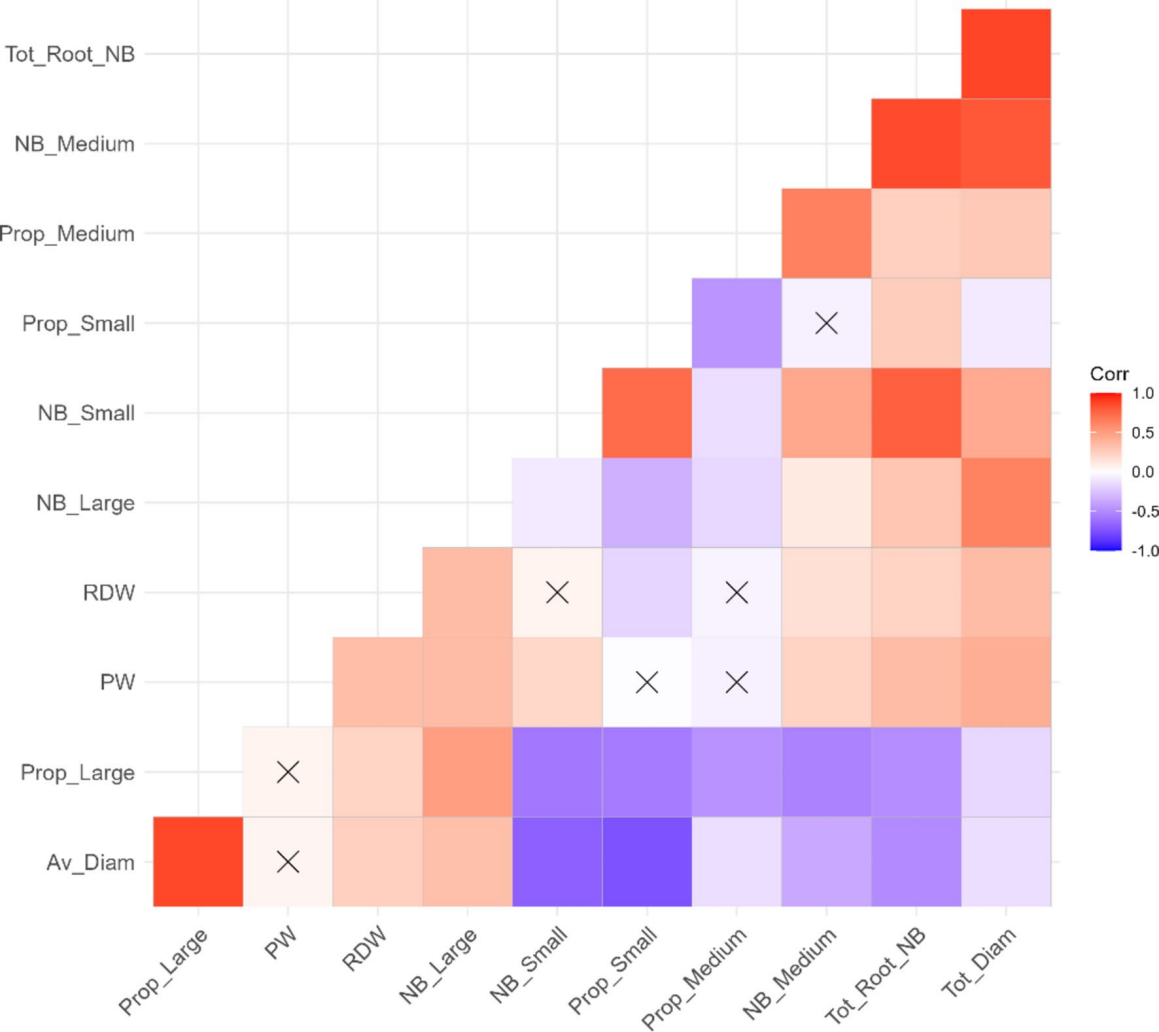
Correlation matrix for root-related traits, based on a Pearson correlation matrix for all root-related traits. Crosses indicate a non-significant correlation and the color indicates the nature of the correlation, with positive correlations shown in red and inverse correlations in blue. The intensity of the hue indicates the strength of the correlation (color figure online)

PW, SD and RSD depended on shoot sampling before grafting, but significant correlations were observed between plant weight and all root-related traits other than the proportions of each class of root (Fig. 4). Positive correlations were observed between Tot_Root_NB and all traits other than Av_Diam and Prop_Large (inversely correlated with Tot_Root_NB). Av_Diam and Prop_Large were inversely correlated with all root-related traits.

### GWAS on root-related traits

The BLUE values were used as phenotypic values for GWAS. Plant weight, year of experimentation and genotype had significant effects in the model for all traits except plant weight for Prop_Medium (Table S3).

GWAS identified 11 markers associated with the Av_Diam of roots (located on chromosomes 8, 10, 17, and 18), Tot_Root_NB (chromosome 5), NB_Small (chromosomes 1, 9, 13, and 17), and NB_Medium (chromosomes 5 and 14) (Table 2). These markers explained between 0.4% and 25.1% of the trait variance. The chr5_19758975 marker was identified for two traits: Tot_Root_NB and NB_Medium.

**Table 2:** Significant markers list (Chr indicates the chromosome, Effect is the intercept calculated in BLINK using the marker as a fixed effect in a linear model, *r*^2^ was calculated in BLINK using the marker as a random effect in a linear model, Maf is the minor allele frequency; genes were obtained with the annotated sequence of *V. berlandieri*, and functions were suggested in UniProt)

Chr	Position	Trait	Effect	*r*^2^ (%)	Maf	*P* value	Size (bp)	Gene	Function	Source
chr8	3,205,879	Average diameter	−0.5	1.7	0.27	1.64E−07	6606	Vitvi08g02318	PREDICTED: UPF0481 protein At3g47200-like Integral component of membrane	IEA:UniProtKB-KW
chr10	24,863,208	Average diameter	2.1	25.1	0.06	2.45E−16	2902	Vitvi10g02297	Unknown	
chr17	4,986,873	Average diameter	0.2	0.9	0.38	5.55E−07	2356	Vitvi17g00422	Strictosidine synthase	IEA:EnsemblPlants
chr18	13,881,469	Average diameter	1.9	1.8	0.25	5.96E−08	9876	Vitvi19g00545; Vitvi18g01271; Vitvi18g01272; Vitvi18g01273	Unknown	
chr5	19,758,975	Total root number	−0.3	0.4	0.12	1.79E−08	855	Vitvi05g01219; Vitvi05g02076	GTPase activity; unknown	IBA:GO_Central
chr1	2,250,037	Number of small roots	0.1	1.4	0.11	6.44E−10	7808	Vitvi01g01633; Vitvi01g01632; Vitvi01g01631	Unknown	
chr9	18,214,759	Number of small roots	0	0.6	0.06	3.78E−11	860	Vitvi09g00521	Metal ion binding	IEA:EnsemblPlants
chr13	8,270,412	Number of small roots	−7	1.0	0.14	9.03E−12	1068	Vitvi13g00728	UMP kinase activity	IBA:GO_Central
chr17	4,296,526	Number of small roots	0.9	8.5	0.17	1.49E−10	2356	Vitvi17g00360; Vitvi17g00361	Transcription regulator; unknown	IBA:GO_Central
chr5	19,758,975	Number of medium-sized roots	−0.6	4.3	0.12	4.85E−08	855	Vitvi05g01219; Vitvi05g02076	GTPase activity; unknown	IBA:GO_Central
chr14	21,295,561	Number of medium-sized roots	− 2.5	6.0	0.09	1.28E−07	1852	Vitvi14g01232	Nuclear organization	IEA:EnsemblPlants

**Fig. 5:**
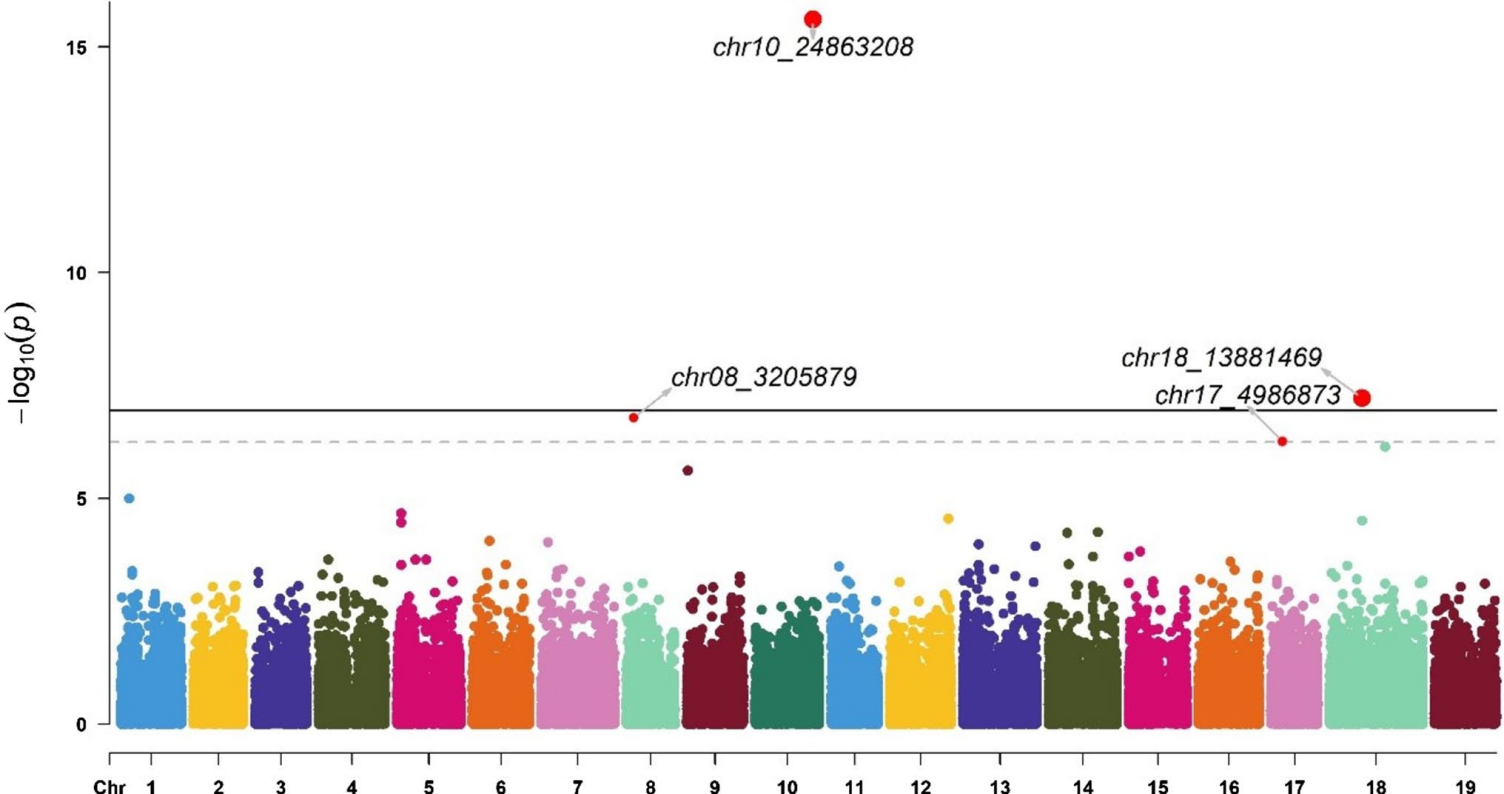
Manhattan plot for SNP associations with mean root diameter (Av_Diam). The thresholds were calculated with the Bonferroni method for *α* = 0.05 (dashed line) and 0.01 (solid line). Significant signals are indicated by a small red dot for *α* = 0.05 and a larger red dot for *α* = 0.01. The corresponding QQ plot is presented in Figure S3A (color figure online)

The chr8_3205879 and chr17_4986873 markers were significant for the Av_Diam (Fig. 5) and explained 1.7% and 0.9% of the trait variance, respectively. The other two significant markers, chr10_24863208 and chr18_13881469, explained 25.1% and 1.8% of the trait variance, respectively. The chr8_3205879 marker was linked to the Vitvi08g02318 gene, chr10_24863208 was linked to Vitvi10g02297, chr17_4986873 was linked to Vitvi17g00422, and chr18_13881469 was linked to Vitvi19g00545, Vitvi18g01271, Vitvi18g01272, and Vitvi18g01273 (Table 2).

**Fig. 6:**
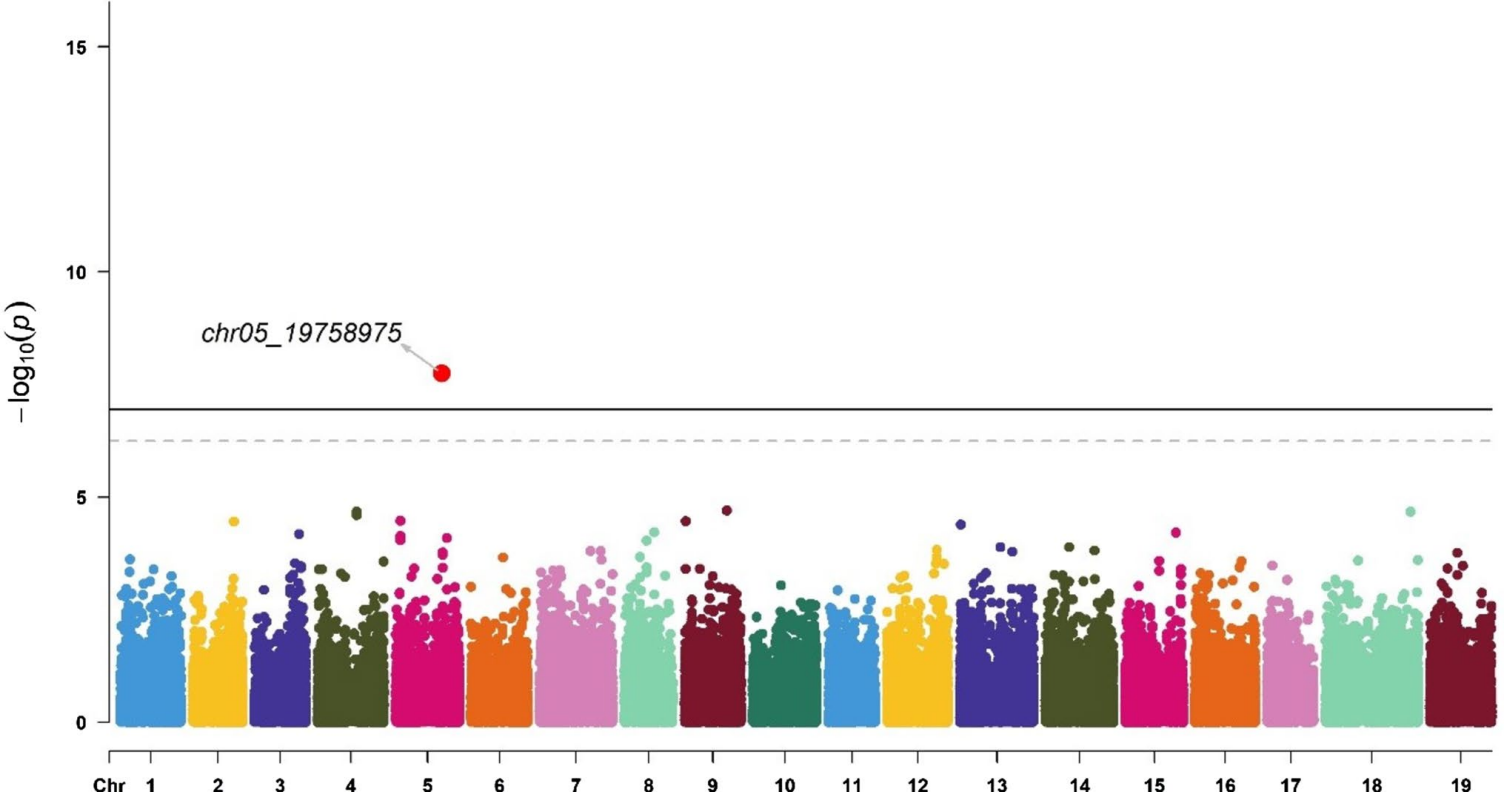
Manhattan plot for SNP associations with total root number (Tot_Root_NB). The thresholds were calculated with the Bonferroni method for *α* = 0.05 (dashed line) and 0.01 (solid line). Significant signals are indicated by a small red dot for *α* = 0.05 and a larger red dot for *α* = 0.01. The corresponding QQ plot is presented in Figure S3B (color figure online)

**Fig. 7:**
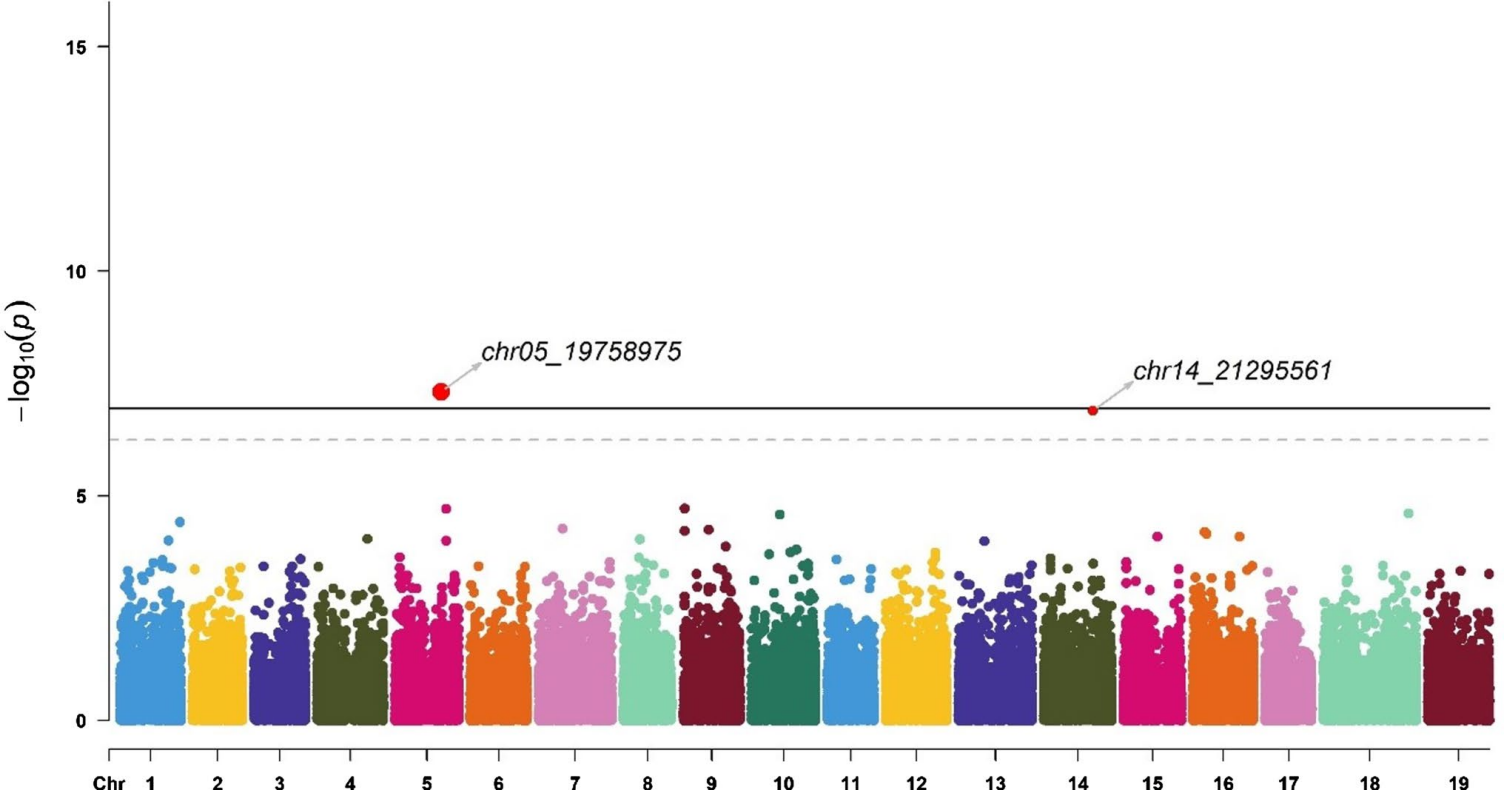
Manhattan plot for SNP associations with the number of medium-sized roots (NB_Medium, diameter from 1 to 2 mm). The thresholds were calculated with the Bonferroni method for *α* = 0.05 (dashed line) and 0.01 (solid line). Significant signals are indicated by a small red dot for *α* = 0.05 and a larger red dot for *α* = 0.01. The corresponding QQ plot is presented in Figure S3D (color figure online)

One marker, chr5_19758975, was identified as significant for both Tot_Root_NB (Fig. 6; Table 2) and NB_Medium (Fig. 7), explaining 0.4% and 4.3% of the variance, respectively, for these traits. It was linked to the genes Vitvi05g01219 and Vitvi05g02076 (Table 2). The chr14_21295561 marker was also found to be significant for NB_Medium, accounting for 6.0% of the variance for this trait and linked to the Vitvi14g01232 gene (Table 2).

**Fig. 8:**
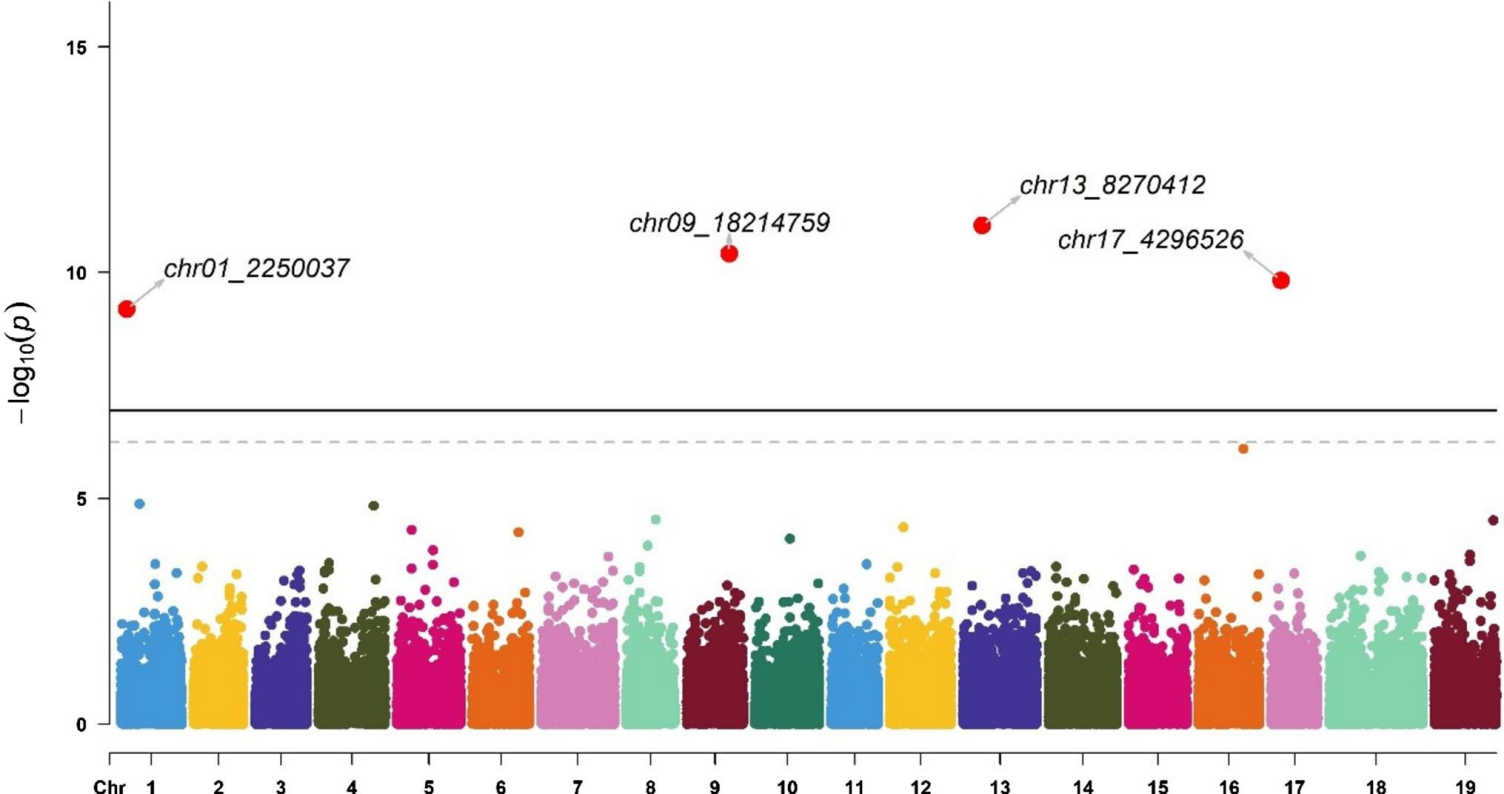
Manhattan plot for SNP associations with the number of small roots (NB_Small, diameter < 1mm). The thresholds were calculated with the Bonferroni method for α = 0.05 (dashed line) and 0.01 (solid line). Significant signals are indicated by a small red dot for α = 0.05 and a larger red dot for α = 0.01. The corresponding QQ plot is presented Figure S3C (color figure online)

NB_Small was significantly associated with chr1_2250037, chr9_18214759, chr13_8270412, and chr17_4296526 (Fig. 8; Table 2) with *r*^2^ = 1.4%, 0.6%, 1.0%, and 8.5%, respectively. The chr1_2250037 marker was linked to Vitvi01g01633, Vitvi01g01632, and Vitvi01g01631 (Table 2). The chr9_18214759 marker was linked to Vitvi09g00521, chr13_8270412 was linked to Vitvi13g00728, and chr17_4296526 was linked to Vitvi17g00360 and Vitvi17g00361 (Table 2).

**Fig. 9:**
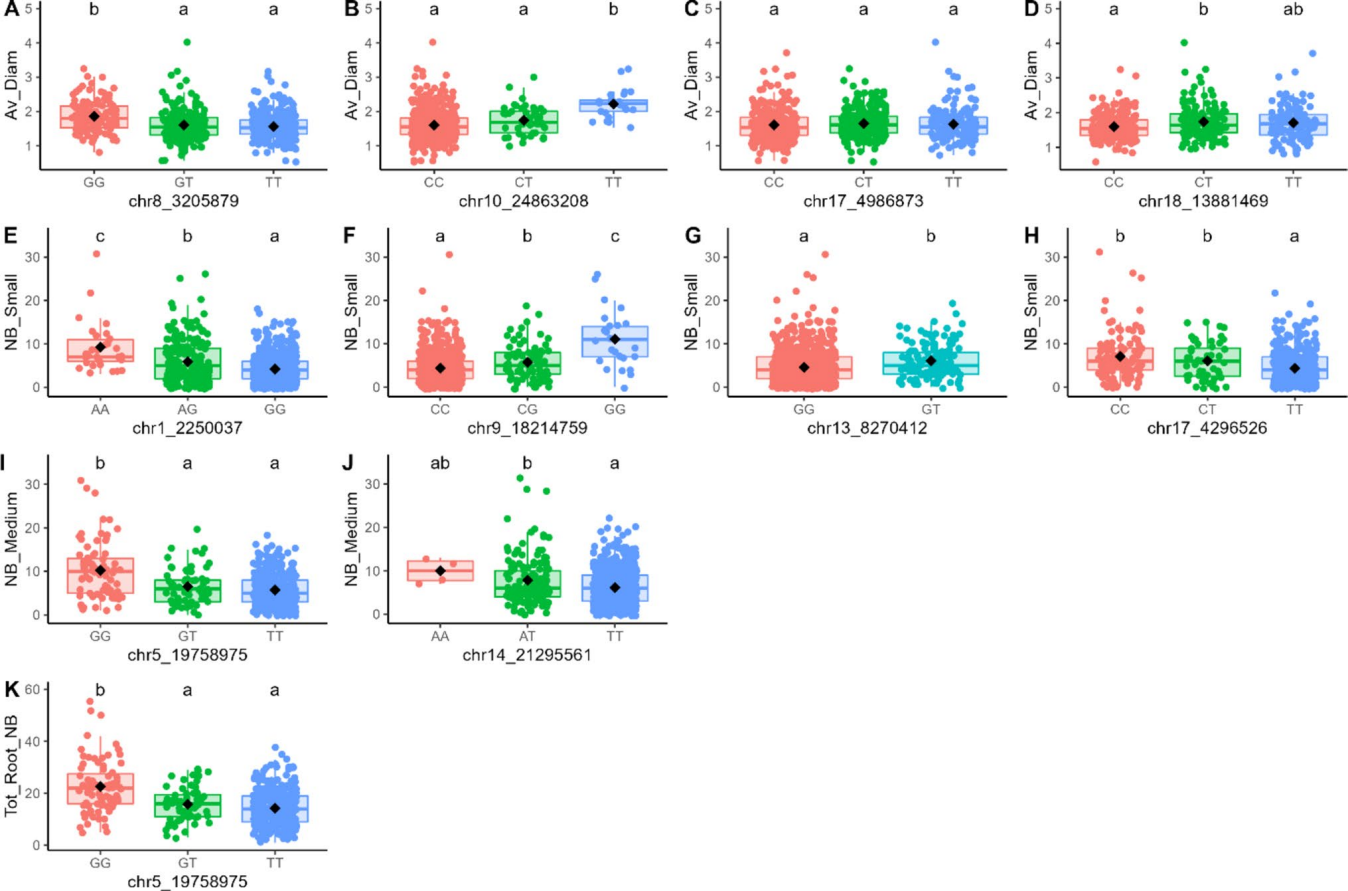
Boxplots of marker effects on root-related traits: chr17_4986873 (**A**), chr8_3205879 (**B**), chr10_24863208 (**C**), and chr18_13881469 (**D**) for mean root diameter (Av_Diam); chr5_19758975 (**E**) for total root number (Tot_Root_NB), chr1_2250037 (**F**), chr13_8270412 (**G**), chr17_4296526 (**H**), and chr9_18214759 (**I**) for the number of small roots (NB_Small, diameter < 1 mm), and chr14_21295561 (**J**) and chr5_19758975 (**K**) for the number of medium roots (NB_Medium, 1 mm > diameter < 2 mm) (color figure online)

For each marker, we explored the effects of each allele in the homozygous and heterozygous states (Fig. 9).

### Root-related trait performances

Commercial rootstocks are widely used in vineyards and were used as controls for favorable root-related traits in comparisons with *V. berlandieri* genotypes. The commercial rootstocks had higher RDW, RN and RSD values than the *V. berlandieri* genotypes. However, a few *V. berlandieri* genotypes had similar values to the commercial rootstocks for these traits in both years (Figure S1). Commercial rootstocks displayed greater variability for RDW, Tot_Root_NB, Av_Diam, NB_Small, and NB_Medium in 2021 than in 2020 (Table S4), probably due to the addition of genotype 5BB to the pool for the second year of the experiment. However, Tot_Diam variability was lower in 2021. Commercial rootstocks performed well, with high values of RDW, Tot_Root_NB, and Tot_Diam, over the two years of experiment (Figure S1 and S2).

The commercial rootstocks (used as additional individuals) had similar coordinates in the two years, with 110R and Börner located close together on the graph (Fig. 3). 5BB was present only in the 2021 panel and can be distinguished by its high RN. The 26,186, 25,436, and 24,894 genotypes stood out on the individual PCA graph (Fig. 3) because they had similar extreme coordinates to the controls on the PCA, in both years of the experiment.

## Discussion

A wild *Vitis* population (*V. berlandieri*) relevant for rootstock breeding was evaluated with two independent sets of plants for root system phenotypes after grafting with the Riesling variety. This population displayed considerable genetic variability for root-related traits. We also performed a GWAS for root-related traits on the *V. berlandieri* population. Significant QTLs were identified for four root-related traits: four for mean diameter, with one marker explaining 25.1% of the trait variance, one for the total number of roots, four for the number of *small* roots, with one marker explaining 8.5% of the trait variance, and two for the number of *medium* roots. We then compared the root system profiles of the *V. berlandieri* population with those of commercial rootstocks, to identify promising genotypes for breeding.

### Genetic variability of root-related traits

Root system architecture is of considerable importance, given its contribution to plant productivity and adaptation. However, plant root systems are difficult to phenotype, resulting in a larger number of studies for annual crops, which have smaller root systems, than for perennials. Root traits have been shown to have a moderate-to-high heritability and coefficient of variation in cotton (Cui et al. 2022) and in several cereal crops, including maize seedlings (Pace et al. 2015; Sanchez et al. 2018), barley (Reinert et al. 2016; Abdel-Ghani et al. 2019), and rice (Courtois et al. 2013; Biscarini et al. 2016; Phung et al. 2016).

Our results were consistent with these previous studies. Root diameter and root number were highly variable and had a moderate-to-high heritability. Tandonnet et al. (2018) obtained similar results for grapevine root section (related to root diameter) and root number (H^2^ = 0.64 and 0.7 respectively) in a *V. vinifera cv.* Cabernet-Sauvignon × *V. riparia cv.* Gloire de Montpellier progeny. Root diameter is related to root function, with thicker roots more involved in transport and storage and representing a long-term investment for the plant (Comas et al. 2010). In rice, thick roots established before drought stress events improve the drought tolerance of the plants (Price and Courtois 1999; Gowda et al. 2011). Conversely, fine roots are involved in the absorption of water and nutrients and represent a short-term investment for the plant. Moreover, root diameter is also related to root hydraulic conductivity (Rieger and Litvin 1999) and mycorrhization capacity (Peat and Fitter 1993), which can affect plant physiology, production and adaptation (Smith and Read 2010). The genetic variability observed for root-related traits in the *V. berlandieri* population may, thus, be correlated with other traits of interest, such as those mentioned above.

In this study, the root system developed from a piece of wood (after grafting), leading to a homorhizic root system architecture composed of adventitious roots initiated from the node of the rootstock. Each root beginning at the node is considered to be a primary root. We studied plants growing on a homogeneous substrate in pots, to limit the impact of soil chemical and physical variability on the growth and development of the root system (Seguin 1972). However, this made it difficult to study root architecture traits, such as angles, density and length. The moderate-to-high variability and heritability of root-related traits observed in this study suggested that it would be worth performing a GWAS for these traits.

### GWAS for root-related traits

We used the BLINK (Bayesian information and linkage disequilibrium iteratively nested keyway) model, which has been shown to have the best performance for detecting significant markers in GWAS (M. Huang et al. 2018).

Root systems have mostly been studied in annual crops, and GWAS has identified markers involved in the determinism of root-related traits principally in cereals, such as maize (Hochholdinger and Tuberosa 2009; Pace et al. 2015; Zaidi et al. 2016; Bray and Topp 2018; Sanchez et al. 2018; Zheng et al. 2020), rice (Courtois et al. 2013; Biscarini et al. 2016; Phung et al. 2016; Kadam et al. 2017; Li et al. 2017; Zhao et al. 2018), wheat (Ayalew et al. 2018; Alahmad et al. 2019; Beyer et al. 2019) and barley (Reinert et al. 2016; Abdel-Ghani et al. 2019). To our knowledge, no previous study has ever been performed on woody perennial grafted plants.

Our GWAS identified 11 markers associated with root-related traits explaining 0.4–25.1% of the variance for individual traits. The proportion of the variance explained by the detected markers was higher in this study than previously reported for cereal crops. Most of the markers were linked to genes of unknown function (Table 2), particularly those explaining more than 25% of the variance for mean diameter. However, chr5_19758975, a marker identified for the total number of roots and the number of medium-sized roots, was linked to Vitvi05g01219, encoding a protein potentially involved in GTPase activity. The chr8_3205879 marker associated with mean diameter was predicted to associated with a gene encoding a protein resembling At3g47200 (Dunkley et al. 2006), which is an integral membrane component. The chr9_18214759 marker was linked to the Vitvi09g00521 gene potentially involved in metal ion binding (Johnson et al. 2005), potentially accounting for the limestone tolerance of the *V. berlandieri* genetic background. The chr13_8270412 marker was linked to the Vitvi13g00728 gene encoding a protein with UMP kinase activity. The chr14_21295561 marker was linked to the Vitvi14g01232 gene involved in nuclear organization (Sajiki et al. 2009). The markers detected on chromosome 17 (chr17_4296526 associated with NB_Small and chr17_4986873 associated with Av_Diam) were linked to the genes Vitvi17g00360 and Vitvi17g00422, respectively. Vitvi17g00360 encodes a transcription regulator, whereas Vitvi17g00422 encodes a protein potentially involved in strictosidine synthesis, which is involved in more than 1000 indole alkaloid pathways (Kutchan 1993). Alkaloids are involved in plant protection against diverse pressures and are present at high concentrations in flowering plants (Sumner et al. 2003). They act as defense chemicals in *Catharanthus roseus* (Luijendijk et al. 1996) and are involved in various pathways in *Arabidopsis thaliana* (defense, drugs for human diseases) (Kibble et al. 2009). One quantitative genetic study carried out on a *Vitis vinifera* cv. Cabernet-Sauvignon × *V. riparia* cv. Gloire de Montpellier progeny based on 212 microsatellite markers has revealed genetic regions correlated with root-related traits (Tandonnet et al. 2018). Similarly to our results, QTL were found on linkage groups 5 and 9 but the large confidence intervals of these markers (40 to 56 cM) made it difficult to compare with our results.

Blois et al. (2023) detected markers associated with environmental variability in the same wild *V. berlandieri* population. Given the short LD decay observed (2.2 kb) in the population, these markers were probably not linked to the markers detected in this study. However, the functions of the genes potentially linked to significant markers were similar. For instance, the chr09_18214759 (this study) and chr07_3341495 (Blois et al. 2023) markers were linked to the Vitvi09g00521 and Vitvi01g01826 genes, respectively, both of which encode proteins involved in metal ion binding. Moreover, the chr15_1889550 marker was linked to the Vitvi15g01070 gene encoding a protein involved in the iron pathway. These genes should be explored in greater depth, because they may account for the outstanding tolerance of limestone conditions observed in this species (Galet 1988).

Given the role of the root system in plant productivity and stress tolerance (Meister et al. 2014; Maqbool et al. 2022), the markers explaining a large proportion of trait variance (*r*^2^ = 25.1% on chr10_24863208 for Av_Diam and *r*^2^ = 8.5% on chr17_4296526 for NB_Small,) should be investigated in greater depth (in a quantitative genetic study with a pedigree population, for example) with a view to inclusion in grapevine rootstock breeding programs.

### Root-related trait performances

In grafted cultivated plants, such as fruit trees, grapevine, and other species, including tomatoes, the root system genotype differs from the scion genotype. It is therefore possible to improve the root system of the plant directly, improving the biotic and abiotic tolerance of the plant, without the need to modify the scion (Marguerit et al. 2012; Tamura 2012).

The commercial rootstocks used have been reported to provide vigor to the scion, together with tolerance to drought, limestone conditions and phylloxera, all of which are parameters of interest for grapevine rootstocks. Root system profiles have a strong impact on drought tolerance and nutrient capture (Lynch 1995). The drought tolerance of commercial rootstocks is considered very high for 110R, high for Börner, moderate-to-high for SO4 and low for 5BB. The relationships between root-related traits and drought tolerance require further exploration. The selection of root system traits of interest is complex, because it depends on environmental conditions, soil properties, plant species, and cultural practices (Lynch 2018). Ideotypes are then explored to identify the root system profiles suitable for the broadest range of environmental conditions. The *steep*, *cheap* and *deep* root system profile has been proposed by Lynch, (2013), in which roots grow deeply but with the lowest “carbon cost” possible. However, this profile was proposed for annual crops, and ideotypes may be very different for perennials, in which the target may also be to invest more carbon in root system growth and development so as to obtain a well-established root system. This would enable the plant to increase the volume of soil explored and to gain access to more water resources in conditions of water deficit. Given the difficulty of selecting root system ideotypes, we based our performance criteria on the root system profiles of commercial rootstocks, which had large numbers of roots of evenly balanced diameters, resulting in high total root diameters. The widespread use of these commercial rootstocks reflects their good performance in the field. We therefore assumed that they perform better than other rootstocks in the field due to their specific root system profiles. Then, *V. berlandieri* genotypes with root system profiles similar to those of commercial rootstocks (24,894, 25,436 and 26,186) therefore constitute promising candidates for use as parental material in breeding programs. It should also be borne in mind that we measured root traits at a juvenile stage (1 year), and that these traits might not be maintained at later stages in this perennial plant. Rootstocks can have a major effect on the physiological processes in scions (Gregory et al. 2013). It is, therefore, very important to test these genotypes in the grafted state in field conditions, to characterize their tolerances of limestone conditions and drought. However, until now the genetic diversity of rootstock has not been explored by species. Our work constitute the first exploration of the genetic determinism of phenotypic traits in a grapevine population from a natural habitat. These results have shown the interest of dissecting the overall genetic diversity from rootstock species in order to exploit the full potential on each American species, which could allow adapting grapevine in the context of climate change.

## Conclusion

Our results highlight the considerable genetic variability of root-related traits in a wild *V. berlandieri* population and the moderate-to-high heritability of these traits. Moreover, we performed a genome-wide association study for root-related traits, which identified 11 markers associated with these traits. Two of these markers explained a large proportion of the trait variance, suggesting that they could be used in marker-assisted selection, to facilitate the breeding of improved rootstocks. A few wild genotypes had performances similar to those of widely used commercial rootstocks. However, these genotypes would need to be characterized for the other agronomic traits important in grapevine rootstocks, such as drought tolerance, limestone tolerance and vigor in field conditions. The genotypes identified may be outstanding candidates for use in breeding and a field experiment is currently being set up in Bordeaux to assess their agronomic performances.

### Supplementary Information

Below is the link to the electronic supplementary material.

Supplementary file1 (PDF 854 KB)

## Declarations

### Data availability

Data for this study are available at: V. berlandieri reference genome: NCBI, BioProject ID: PRJNA886625. V. berlandieri population GBS data: NCBI, BioProject ID: PRJNA886619. Phenotypic data: Available on request to the corresponding author.
