# Supplementary material for: Dissecting the genetic architecture of root-related traits in a grafted wild Vitis berlandieri population for grapevine rootstock breeding
Source: Theor Appl Genet. 2023 Oct 14;136(11):223. doi: 10.1007/s00122-023-04472-1 (PMC10576685; doi:10.1007/s00122-023-04472-1)
Supplement: Supplementary file 1 — Supplementary file1 (PDF 854 KB) [file 122_2023_4472_MOESM1_ESM.pdf]

Supplemental data

Table S1: Number of replicates per genotype in 2020, 2021 an their combination (Overall)

	2020	2021	Overall
1 replicate	47	63	48
2 replicates	38	34	32
3 replicates	33	23	26
4 replicates	27	14	28
5 replicates	36	10	21
6 replicates	0	0	17
7 replicates	0	0	15
8 replicates	0	0	13
9 replicates	0	0	5
10 replicates	0	0	6
Total	181	144	211

Table S2: General root trait statistics for the *V. berlandieri* population

	2020				2021			
	**Min**	**Max**	**Mean**	**StDev**	**Min**	**Max**	**Mean**	**StDev**
RDW (g)	0.4	10.9	3.4	1.7	0.1	16.7	5.0	3.1
PW (g)	15.9	47.7	27.6	5.5	15.8	49.5	27.0	5.6
SD (mm)	3.2	7.3	4.9	0.7	2.3	6.7	4.5	0.7
RSD_1 (mm)	5.2	10.6	7.2	0.9	5.0	11.2	7.3	0.9
RSD_2 (mm)	6.0	11.8	8.4	1.0	5.9	11.6	8.2	0.9
Tot_Root_NB	2.0	55.0	17.2	7.7	1.0	35.0	13.0	6.5
Tot_Diam (mm)	4.8	71.7	26.4	10.1	3.5	46.4	19.6	8.2
Av_Diam (mm)	0.7	3.2	1.6	0.4	0.5	4.0	1.7	0.5
NB_Small	0.0	26.0	5.1	4.1	0.0	31.0	4.4	4.0
NB_Medium	0.0	31.0	7.6	4.7	0.0	19.0	5.0	3.6
NB_Large	0.0	14.0	4.5	2.3	0.0	11.0	3.6	2.1
Prop_Small	0.0	0.8	0.3	0.2	0.0	0.9	0.3	0.2
Prop_Medium	0.0	0.9	0.4	0.2	0.0	1.0	0.4	0.2
Pop_Large	0.0	0.9	0.3	0.2	0.0	1.0	0.3	0.2

Table S3: Best linear unbiased estimates (BLUE) model output. The significance of the factors used in the BLUE model are indicated in the Pr(>F) column. The factors indicated are the year of the experiment, plant weight (PW) and genotype.

Trait	Factor	Pr(>F)
RDW	Year	< 2.2e-16
	PW	< 2.2e-16
	Genotype	< 2.2e-16
Tot_Root_NB	Year	< 2.2e-16
	PW	1.62e-15
	Genotype	< 2.2e-16
Tot_Diam	Year	< 2.2e-16
	PW	< 2.2e-16
	Genotype	< 2.2e-16
Av_Diam	Year	0.01
	PW	4.8e-04
	Genotype	< 2.2e-16
NB_Small	Year	0.02
	PW	0.05
	Genotype	< 2.2e-16
NB_Medium	Year	< 2.2e-16
	PW	3.8e-08
	Genotype	< 2.2e-16
NB_Large	Year	1.7e-07
	PW	3.1e-16
	Genotype	1.2e-14
Prop_Small	Year	0.05
	PW	0.04
	Genotype	9.6e-11
Prop_Medium	Year	1.e-07
	PW	0.6
	Genotype	2.4e-06
Prop_Large	Year	5.9e-04
	PW	5.1e-03
	Genotype	< 2.2e-16

Table S4: General root trait statistics for commercial rootstocks (110R, SO4 and Borner in 2020 and 2021, with 5BB added to the pool in 2021).

	2020				2021			
	**Min**	**Max**	**Mean**	**StDev**	**Min**	**Max**	**Mean**	**StDev**
RDW (g)	4.3	11.1	6.5	1.8	8.1	16.4	12.7	2.3
PW (g)	34.8	51.7	40.1	4.3	25.0	41.9	34.3	4.5
SD (mm)	4.5	6.7	5.7	0.8	3.9	6.5	5.1	0.7
RSD_1 (mm)	7.7	10.4	9.0	0.8	6.8	9.6	8.3	0.7
RSD_2 (mm)	8.8	11.6	10.1	0.8	8.1	11.5	9.7	0.9
Tot_Root_NB	17.0	45.0	28.6	7.8	16.0	52.0	30.3	10.2
Tot_Diam (mm)	28.5	69.7	45.5	10.8	24.8	49.6	37.2	7.2
Av_Diam (mm)	1.4	1.8	1.6	0.1	0.9	1.8	1.3	0.2
NB_Small	3.0	18.0	7.9	3.6	4.0	28.0	13.2	7.4
NB_Medium	7.0	27.0	13.1	5.1	5.0	24.0	12.7	5.8
NB_Large	4.0	12.0	7.7	2.2	1.0	8.0	4.4	2.1
Prop_Small	0.2	0.4	0.3	0.1	0.1	0.6	0.4	0.1
Prop_Medium	0.4	0.7	0.5	0.1	0.3	0.6	0.4	0.1
Pop_Large	0.1	0.4	0.3	0.1	0.0	0.4	0.2	0.1

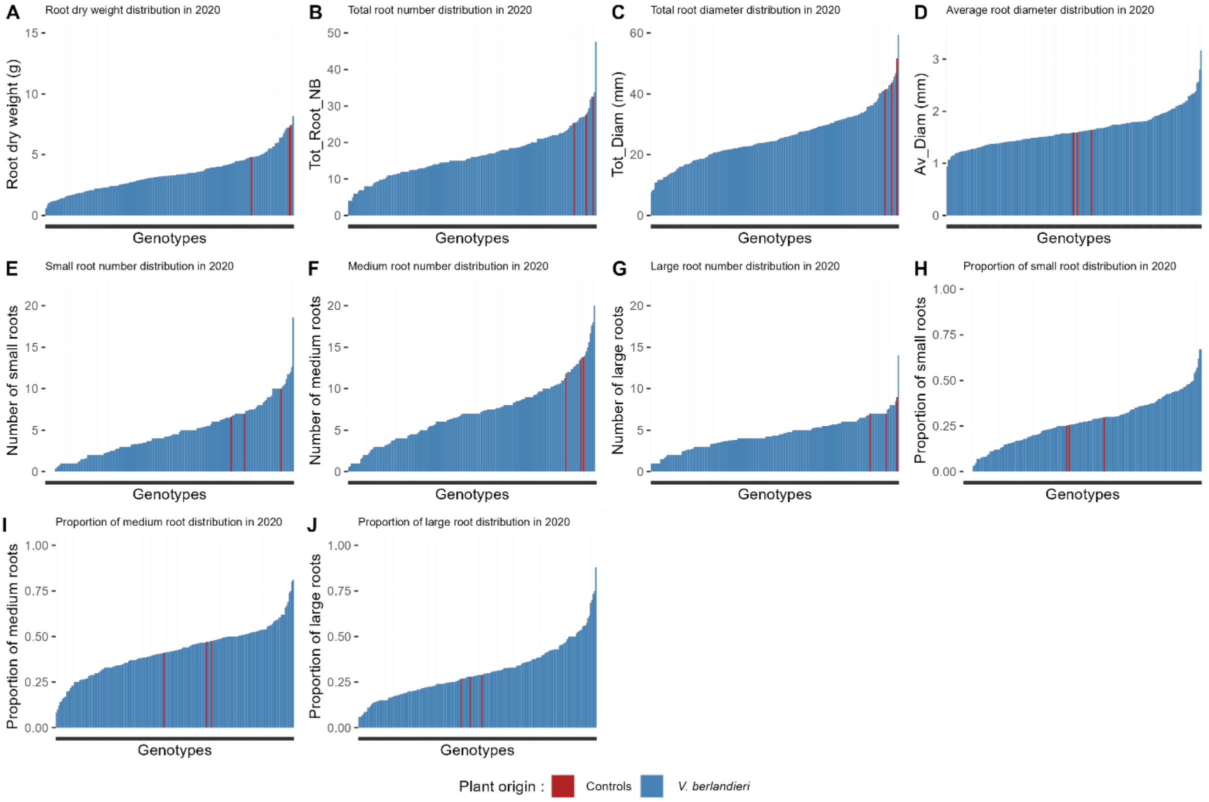

Figure S1: Distributions of root-related traits measured in 2020. For each trait, red lines indicate the positions of the commercial rootstocks in the distribution (Borner, 110R, and SO4). The traits shown are root dry weight (A), the total root number (Tot_Root_NB, B), the total diameter (Tot_diam, C), the average diameter (Av_Diam, D), the number of small roots (diameter < 1 mm, E), the number of medium-sized roots (1 mm < diameter < 2 mm, F), the number of large roots (diameter > 2 mm, G), the proportion of small roots (H), the proportion of medium roots (I), and the proportion of large roots (J).

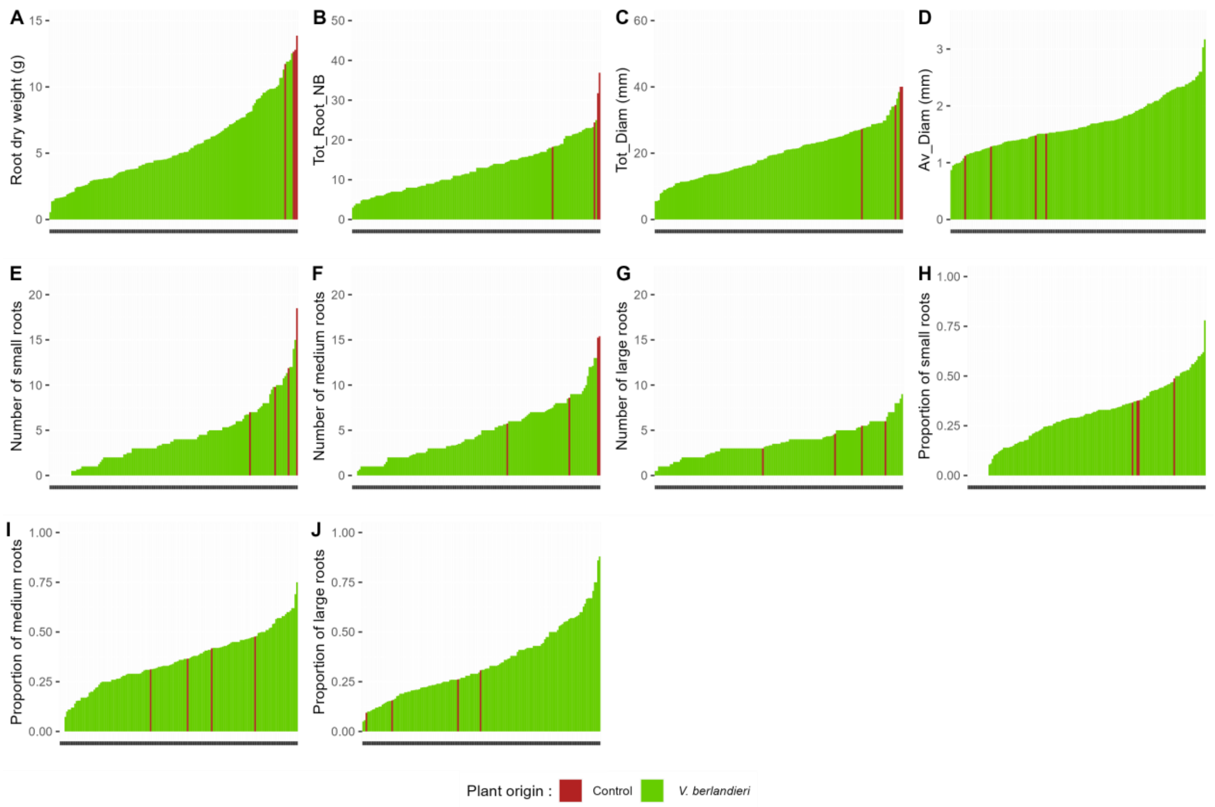

Figure S2: Distributions of root-related traits measured in 2021. For each trait, red lines indicate the positions of the commercial rootstocks in the distribution (Borner, 110R, SO4, and 5BB). The traits shown are root dry weight (A), the total root number (Tot_Root_NB, B), the total diameter (Tot_diam, C), the average diameter (Av_Diam, D), the number of small roots (diameter < 1 mm, E), the number of medium-sized roots (1 mm < diameter < 2 mm, F), the number of large roots (diameter > 2 mm, G), the proportion of small roots (H), the proportion of medium roots (I), and the proportion of large roots (J).

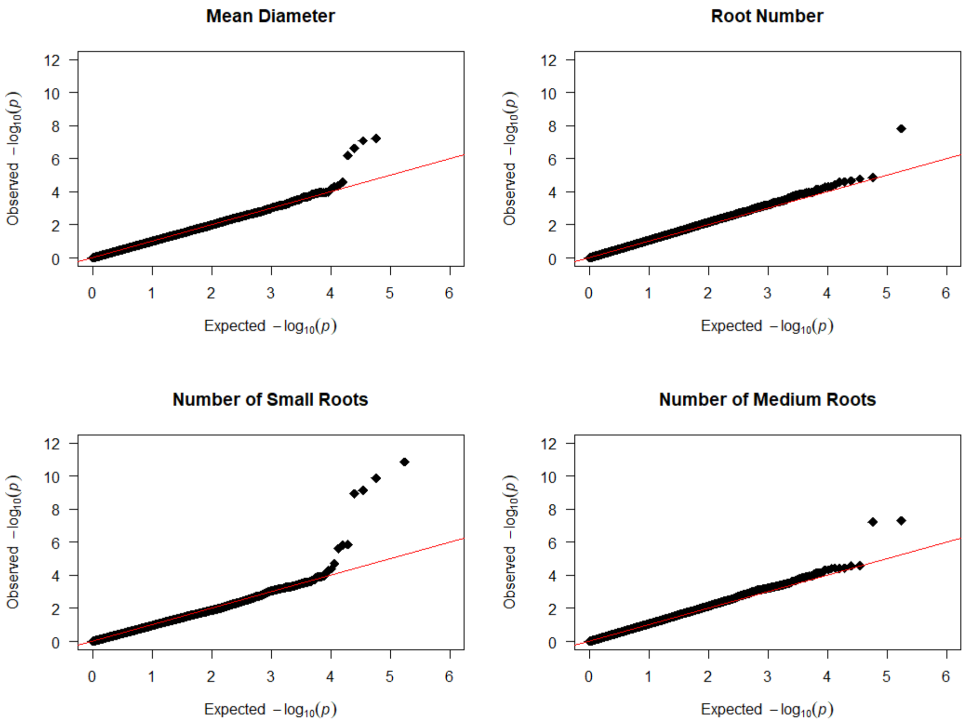

Figure S3: QQ plot, indicating the distributions of theoretical and observed *p*-values in the genome-wide association study for root mean diameter (A), total root number (B), the number of small roots (C), and the number of medium-sized roots (D).
